# IL-6 Improves the Nitric Oxide-Induced Cytotoxic CD8+ T Cell Dysfunction in Human Chagas Disease

**DOI:** 10.3389/fimmu.2016.00626

**Published:** 2016-12-23

**Authors:** Liliana Maria Sanmarco, Laura Marina Visconti, Natalia Eberhardt, Maria Cecilia Ramello, Nicolás Eric Ponce, Natalia Beatriz Spitale, Maria Lola Vozza, Germán Andrés Bernardi, Susana Gea, Angel Ramón Minguez, Maria Pilar Aoki

**Affiliations:** ^1^Centro de Investigaciones en Bioquímica Clínica e Inmunología CIBICI-CONICET, Facultad de Ciencias Químicas, Universidad Nacional de Córdoba, Córdoba, Argentina; ^2^Hospital Nuestra Señora de la Misericordia del Nuevo Siglo, Córdoba, Argentina

**Keywords:** tyrosine nitration, peroxynitrite, CD39, CD73, oxidative stress, *Trypanosoma cruzi* infection

## Abstract

Reactive oxygen and nitrogen species are important microbicidal agents and are also involved in lymphocyte unresponsiveness during experimental infections. Many of the biological effects attributed to nitric oxide are mediated by peroxynitrites, which induce the nitration of immune cells, among others. Our group has demonstrated that nitric oxide is involved in the suppressive activity of myeloid-derived suppressor cells in *Trypanosoma cruzi*-infected mice, with a higher number of CD8+ T cells suffering surface-nitration compared to uninfected controls. Studying the functional and phenotypic features of peripheral CD8+ T cells from chagasic patients and human cells experimentally infected with *T. cruzi*, we found that different regulatory mechanisms impaired the effector functions of T cytotoxic population from seropositive patients. Peripheral leukocytes from chagasic patients showed increased nitric oxide production concomitant with increased tyrosine nitration of CD8+ T cells. Additionally, this cytotoxic population exhibited increased apoptotic rate, loss of the TCRζ-chain, and lower levels of CD107a, a marker of degranulation. Strikingly, IL-6 stimulation of *in vitro*-infected peripheral blood mononuclear cells obtained from healthy donors, blunted *T. cruzi*-induced nitration of CD3+CD8+ cells, and increased their survival. Furthermore, the treatment of these cultures with an IL-6 neutralizing antibody increased the percentage of *T. cruzi*-induced CD8+ T cell nitration and raised the release of nitric oxide. The results suggest that the under-responsiveness of cytotoxic T cell population observed in the setting of long-term constant activation of the immune system could be reverted by the pleiotropic actions of IL-6, since this cytokine improves its survival and effector functions.

## Introduction

CD8+ T cells play a critical role in the immunity against intracellular pathogens, including the protozoan parasite *Trypanosoma cruzi*, the causative agent of Chagas cardiomyopathy (1). Chagas disease is characterized by two distinct phases: the acute phase, which lasts several weeks and is characterized by non-specific symptoms; and the chronic phase, lasting lifelong. The host’s ability to control *T. cruzi* infection is substantial, but not fully effective, since most infected individuals tightly limit parasite numbers but fail to completely clear the infection due to diverse and fascinating immune evasion processes (2). Indeed, parasite persistence at low levels into target cells is the hallmark of the indeterminate or asymptomatic chronic phase (3). Up to 40% of infected individuals develop progressive heart disease leading to an end-stage dilated cardiomyopathy.

One key defense mechanism against *T. cruzi* is exerted by nitric oxide (NO), which is produced by inducible NO synthase, among other enzymes, present in monocytes/macrophages and cardiomyocytes (4, 5). The parasite triggers activation of macrophage NADPH oxidase, resulting in a continuous production of superoxide anion $\left({\text{O}}_{2}^{-}\right)$ and also stimulates infected macrophages to produce different amounts of NO. Despite its importance as a microbicidal agent at high levels, persistent low levels of NO have been involved in the establishment and maintenance of lymphocyte unresponsiveness in different experimental models of parasite infections (6–8). These mechanisms illustrate a clear parasite evasion strategy. Moreover, NO induces apoptosis of many different cell types *in vitro* and *in vivo* (9, 10). We found that IL-6 regulates inflammasome activation and, consequently, IL-1β-induced NO production in a murine model of *T. cruzi* infection. The anti-inflammatory action of IL-6 seems to be central for controlling local and systemic oxidative stress, promoting cellular rescue of apoptosis, and protecting infected IL-6-deficient mice against death (unpublished observation). This could be a novel mechanism that regulates NO release in the setting of *T. cruzi* infection.

The presence of low levels of NO rapidly initiates a reaction with the superoxide anion generating peroxynitrites that induce the nitration of surface proteins on T cells (11, 12). It is widely accepted that reactive nitrogen species (NO and peroxynitrites) contribute to the immunosuppressive attitude of myeloid-derived suppressor cells (MDSCs), a heterogeneous cell population associated with tumors, infectious, and inflammatory diseases. Our group has demonstrated that MDSCs during acute *T. cruzi* experimental infection were able to inhibit T cell proliferation *in vitro* (13). Furthermore, we also observed a higher number of splenic CD8+ T cells suffering surface nitration in infected mice compared to uninfected controls.

Chronic exposure to antigens may cause functional defects in pathogen-specific T cells. It has been reported that individuals with more severe clinical disease have a significantly lower frequency of *T. cruzi*-specific CD8+IFNγ+ T cells than subjects in the asymptomatic stage of the infection (14). This impairment in *T. cruzi*-specific CD8+ T cell responses was associated with an increased frequency of fully differentiated memory cells and an increased rate of apoptosis in the total peripheral CD8+ T cell population. The results reveal a progressive exhaustion in the parasite-specific cytotoxic T cell compartment in patients with long-term *T. cruzi* infection. However, during persistent infection, the chronic exposure to an inflammatory microenvironment may also contribute to the impairment of CD8+ T cell responses, resulting in a less efficient control of the pathogen and promoting its persistence.

These findings prompted us to investigate the frequency and functionality of circulating CD8+ T cells from patients with chronic Chagas disease, with particular focus on the molecular mechanisms triggered by IL-6 associated with cytotoxicity and NO-induced cell death. In agreement with previous reports in seropositive patients with Chagas disease, we found a substantial reduction in the total peripheral T cell compartment at the expense of CD8+ T cells (14). Additionally, we observed increased NO-producing leukocytes concomitant with increased nitration of CD8+ T cells and impaired cytotoxic functionality of T cells. Strikingly, IL-6 prevented the nitration of CD8+ T cells and increased their survival when healthy donor peripheral blood mononuclear cells (PBMCs) were infected *in vitro*. In this context, IL-6 led to a decrease in IL-1β levels. The results suggest that the under-responsiveness of the whole cytotoxic T cell population from chagasic patients could be reverted by the pleiotropic actions of IL-6, since this cytokine functions as a survival factor for CD8+ cells and improves the cytotoxic T cell functionality in the setting of long-term constant activation of the immune system.

## Materials and Methods

### Ethics Statement

Subjects were recruited at the “Hospital Nuestra Señora de la Misericordia” (HNSM) from Córdoba-Argentina. All studies were conducted according to the principles expressed in the Declaration of Helsinki. Signed informed consent was obtained from all donors before inclusion in the study. Venous blood was drawn from 46 non-chagasic and chagasic subjects (age 25–60 years) using a protocol that was reviewed and approved by the Comité Institucional de Ética de la Investigación en Salud del Adulto, Ministerio de Salud de la Provincia de Córdoba (Acta number 194/2014, Argentina). *T. cruzi* infection was determined by a combination of indirect hemagglutination (IHA) and enzyme-linked immunosorbent assay (ELISA) performed in the laboratory of HNSM. Subject positive on these two tests were considered infected. Chronic chagasic patients (*n* = 22) were evaluated clinically and by electrocardiogram and chest X-ray. The uninfected control group (*n* = 24) consisted of age-matched individuals were serologically negative for *T. cruzi*. All donors with chronic or inflammatory pathology or erythrocyte sedimentation rate >30 mm or white blood cells count <4,000 or >10,000/mm^3^ were excluded from the study.

### Blood Collection

Approximately 15 mL of blood was drawn from each individual by venipuncture and placed into heparinized tubes (Vacutainer, BD Bioscience). An aliquot of 50 µL of total peripheral blood was stained for FACS analysis and subjected to ACK lysing buffer to remove erythrocytes. PBMCs were isolated through density gradient centrifugation using Ficoll-Hypaque PLUS (GE Healthcare Bioscience) and resuspended in RPMI 1640 (Gibco) supplemented with 10% heat-inactivated FCS (Natocor).

### *Ex Vivo* Flow Cytometry

Peripheral blood was lysed with ACK lysing buffer to remove erythrocytes, and 01 × 10^6^ cells were blocked with Fc block and stained with anti-human CD3 Alexa 488 or PerCP, anti-human CD4 Alexa 647, anti-human CD8 PECy7, anti-human CD39 biotin and streptavidin APC, anti-human CD73 PE (eBioscience and Biolegend), anti-nitrotyrosine (Sigma-Aldrich), and anti-rabbit Alexa 647 or CD20 PECy7 (Biolegend). The intracellular expression of ζ-chain was analyzed with anti-human TCRζ PE antibody (Beckman Coulter). Stained cells were analyzed by flow cytometry (FACS Canto II, Becton Dickinson), with cellular debris being excluded from the analysis. Data were analyzed using the FlowJo software.

### Cell Viability Measurement

Leukocytes from peripheral blood were stained with anti-human CD3 Alexa 488, anti-human CD4 Alexa 647, anti-human CD8 PECy7 or APCCy7, anti-human CCR7 Alexa 488, anti-human CD45RA PECy7, and labeled with 5 µL of Annexin VPE (BD Pharmingen) for 15 min on ice. Before acquisition, the cells were stained with 7-AAD (BD Bioscience). A minimum of 300,000 events for each condition were analyzed by flow cytometry. Staining of peripheral blood T cells with antibodies to CD45RA and CCR7 reveals four cells subsets: naïve T cells are CD45RA+CCR7+, central memory cells are CD45RA−CCR7+, effector memory (EM) cells are CD45RA−CCR7−, and terminally differentiated effector memory (EMRA) are CD45RA+CCR7− (15).

To determine Bcl-2 expression, PBMCs stained for CD3 PerCP, CD8, CD4, CCR7, and CD45RA surface expression were permeabilized with FOXP3 staining buffer set (eBioscience) and labeled with anti-Bcl-2 rabbit (Cell Signaling) and then with anti-rabbit Alexa 488 or anti-rabbit Alexa 647. Stained cells were analyzed by flow cytometry.

### CD8+ T Cell Functionality

Peripheral blood mononuclear cells were cultured with anti-CD107a PE (Biolegend), monensin, brefeldin A, phorbol 12-myristate 13-acetate (PMA), and ionomycin (Sigma) for 4 h, then stained with anti-IFNγ FITC, anti-TNF APC, and anti-IL-2 APC-Cy7 (Biolegend or eBioscience), and analyzed by flow cytometry.

To evaluate the effect of TCR-dependent activation, PBMCs were cultured with anti-CD3 (1 µg/mL) and anti-CD28 (0.5 mg/mL). After 72 h, PBMCs were cultured with monensin, brefeldin A, and anti-CD107a PE for 6 h and then stained with anti-human CD8 APCCy7, anti-NT rabbit and anti-rabbit Alexa 647, anti-IL-2 PECy7, anti-TNF PerCPCy5.5, or anti-IFNγ PerCPCy5.5.

### CD8+ T Cell Exhaustion

Peripheral blood mononuclear cells were cultured with anti-CD3 (1 µg/mL) and anti-CD28 (0.5 mg/mL). After 72 h, the cells were stained with anti-human CD8 APCCy7, anti-Tim3 PerCPCy5.5, anti-PD1 PECy7, anti-CTLA4 PE, anti-CCR7 Alexa 488, and anti-NT rabbit and anti-rabbit Alexa 647.

### Measurement of Reactive Oxygen and Nitrogen Species

The nitrite/nitrate content, indicative of NO production, was monitored by the Griess reagent assay (16). In 96-well plates, plasma samples were mixed with 50 µL of Griess reagent, consisting of 1% sulfanilamide in 5% phosphoric acid and 0.1% *N*-(1-napthyl) ethylenediamine dihydrochloride [1:1 ratio (vol/vol)], and incubated for 10 min. The change in absorbance was monitored at 545 nm (standard curve, 0–200 µmol sodium nitrite).

The production of ROS and NO was evaluated using the molecular probes: H_2_DCF-DA (10 µM, Invitrogen Inc.) and DAF-FM DA (10 µM, Molecular Probes, Inc.), respectively. All samples were acquired on a FACS Canto II cytometer and then analyzed using the Flow Jo software.

### Quantification of Cytokines

Plasma samples were analyzed for TNF, IL-6, IL-10, IFNγ, and IL-4 by using bead-based immunoassays and flow cytometry, according to the manufacturer’s instructions (LegendPlex-Biolegend). In cultured supernatant, IL-1β levels were measured by Ready-SET-Go ELISA kit from eBioscience.

### Culture of PBMCs and Parasites

Vero cell monolayers were infected with trypomastigote forms of *T. cruzi* Tulahuen strain for 3 h and then washed and maintained in RPMI (Gibco Invitrogen Corporation) at 37°C in a 5% CO_2_ atmosphere. After 7 days, the parasites were collected from the supernatant of infected cells and harvested by centrifugation at 4,400 rpm for 5 min.

Peripheral blood mononuclear cells from non-chagasic donors were cultured with *T. cruzi* Tulahuen trypomastigotes (1:1 rate) for 3 h, then the cells were washed and cultured with recombinant bioactive IL-6 (20 ng/mL) or with anti-IL-6 (2 µg/mL) (Biolegend) or anti-IL-6 plus anti-IL-1β (2 µg/mL), or maintained in medium alone for 48 h. Then, culture supernatants were evaluated for IL-1β/NO levels, and the cells were analyzed for NT staining or NO production by flow cytometry. In addition, peripheral blood from chagasic patients and control donors (250 µL) were cultured with 7,500 trypomastigotes with or without IL-6 (20 ng/mL) or maintained in medium for 24 h and then cells were harvested to evaluate NO and ROS production and NT by flow cytometry.

### Statistical Analysis

Statistical analysis was performed with GraphPad Prism 5.0 software by using parametric or non-parametric paired *t* test according to data distribution. *p* Values <0.05 were considered significant.

## Results

### Chagasic Patients Showed a Lower Number of Peripheral Blood CD8+ T Lymphocytes

A total of 46 peripheral blood samples from chagasic and non-chagasic donors of both sexes, collected from people living in Cordoba (Argentina) were analyzed for *T. cruzi*-specific antibodies by ELISA and IHA (Table 1). The median value for anti-*T. cruzi* antibody titers detected by IHA was 1/256 (local cutoff titer 1/32). Only one patient showed a complete right-bundle branch block, left anterior hemiblock, and left atrial enlargement, clinical features of chagasic heart disease. Cordoba is considered by the Argentine Ministry of Health to be at high risk for vector transmission, since there is a reemergence of this infection route by an increase in house infestation and a high seroprevalence in vulnerable groups (17).

**Table 1:** **Description of subject groups**.

	Non-chagasic donors (*n* = 24)	Chagasic patients (*n* = 22)
**Age (years old)**		
Range	25–60	25–48
Median	30	32
**Gender**		
Female	*n* = 16	*n* = 17
Male	*n* = 8	*n* = 5
**Clinical evaluation**		
Electrocardiographic changes	NE	*n* = 1^a^
Echocardiographic changes	NE	*n* = 1^b^
Chest X-rays abnormalities	NE	*n* = 0

*^a^Complete right-bundle branch block and left anterior hemiblock*.

*^b^Left atrial enlargement*.

*NE, not evaluated*.

Seropositive patients with Chagas disease showed a diminished percentage and absolute number of total peripheral T lymphocytes (CD3+) compared to seronegative donors. Although no differences were observed between non-chagasic and chagasic patients in the percentage and absolute number of T helper cells (CD3+CD4+), the percentage and absolute number of cytotoxic T cells (CD3+CD8+) were significantly lower in seropositive than in seronegative patients (Table 2).

**Table 2:** **Peripheral blood lymphocyte subpopulations**.

	Non-chagasic donors (*n* = 15)	Chagasic patients (*n* = 8)	*p*-Value
**Lymphocyte subsets in peripheral blood**			
CD3+ (%)	58.6 ± 3.9	43.2 ± 5.3	*p* = 0.03*
CD3+/μL (absolute number)	1,139 ± 102	754 ± 135	*p* = 0.04*
CD3+CD4+ (%)	58.7 ± 3.7	57.6 ± 2.0	*p* = 0.84
CD3+CD4+/μL (absolute number)	600 ± 72	535 ± 65	*p* = 0.59
CD3+CD8+ (%)	39.9 ± 3.5	30.74 ± 2.2	*p* = 0.04*
CD3+CD8+/μL (absolute number)	381 ± 56	193 ± 32	*p* = 0.049*
CD20+ (%)	14.1 ± 1.5	12.3 ± 1.6	*p* = 0.44
CD20+/μL (absolute number)	305 ± 40	218 ± 32	*p* = 0.16

*Values are expressed as mean value ± SE*.

*p < 0.05, significant.

No differences were observed in the percentage and absolute number of B lymphocytes (CD20+) between the analyzed groups (Table 2).

### Cytotoxic T Lymphocytes from Chagasic Patients Showed Higher Cell Death and Less Bcl-2 Expression

**Figure 1:**
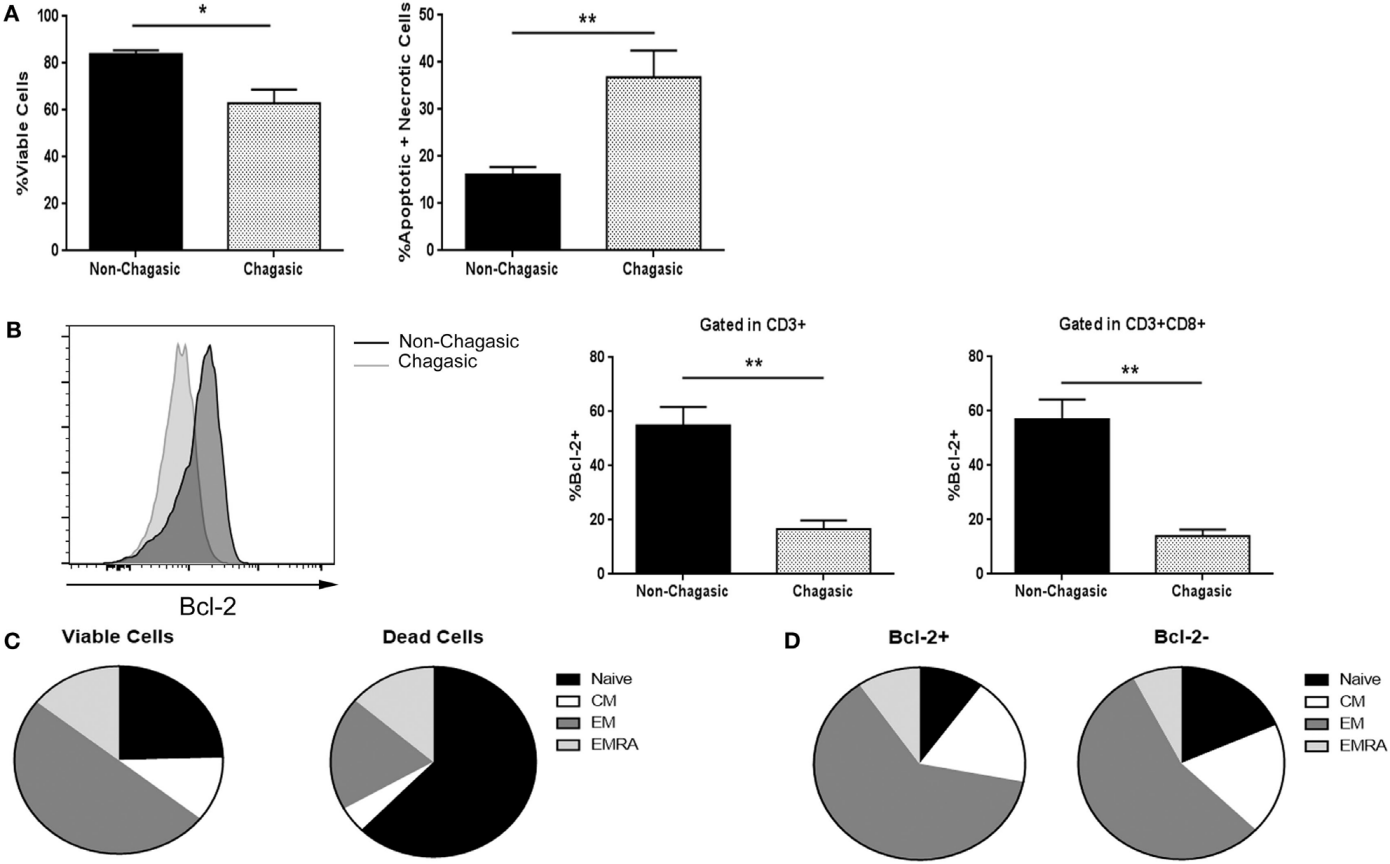
**Cytotoxic T lymphocytes from chagasic patients show higher death rate and less Bcl-2 expression**. **(A)** Percentage of viable and apoptotic + necrotic CD3+CD8+ from chagasic patients (*n* = 5) and non-chagasic donors (*n* = 5) analyzed by Annexin-V and 7AAD staining. **(B)** Representative histogram of Bcl-2 expression in cytotoxic T cells and percentage of T lymphocytes and CD8+ T cells expressing Bcl-2 in chagasic patients (*n* = 5) and in non-chagasic donors (*n* = 5) (*p < 0.05 and **p < 0.01). **(C)** Frequency of naïve, central memory (CM), effector memory (EM), and effector memory RA (EMRA) gated in viable cells (7AAD−) or dead cells (7AAD+) in CD8+ lymphocytes from seropositive patients (*n* = 4) (viable naïve vs. dead naïve *p* = 0.002; viable EM vs. dead EM, *p* = 0.0002). **(D)** Frequency of naïve, CM, EM, and EMRA gated in Bcl-2+ or Bcl-2− in CD8+ cells from chagasic patients (*n* = 4) (naïve Bcl-2+ vs. naïve Bcl-2−, *p* = 0.0123).

Through Annexin V and 7-AAD staining, we found that fresh explanted CD8+ T lymphocytes from chagasic patients showed a diminished percentage of viable cells, and concomitantly, a higher rate of apoptotic and necrotic cells compared to non-chagasic donors (Figure 1A). In agreement, the expression of the anti-apoptotic protein Bcl-2 was significantly higher in cytotoxic lymphocytes from non-chagasic than from chagasic patients (Figure 1B). Among cytotoxic T cells from Chagas patients, naïve CD8 T cells were the phenotype most susceptible to die (viable cells vs. dead cells: 24.53 ± 6.11 vs. 62.48 ± 3.95%). In contrast, EM cytotoxic T cells showed significantly increased survival rate (viable cells vs. dead cells: 49.75 ± 3.06 vs. 19.48 ± 2.45%) (Figure 1C). In accordance, the frequency of naïve cells negative for intracellular Bcl-2 expression was significantly lower than naïve cells positive for the expression of this anti-apoptotic protein (Bcl-2+ vs. Bcl-2−: 9.95 ± 0.87 vs. 17.67 ± 2.07) (Figure 1D). Furthermore, the percentage of EM CD8 T cells was higher in Chagas subjects in comparison with seronegative donors (Figure S1 in Supplementary Material).

### Nitric Oxide-Producing Leukocytes Were Increased in Chagasic Patients

**Figure 2:**
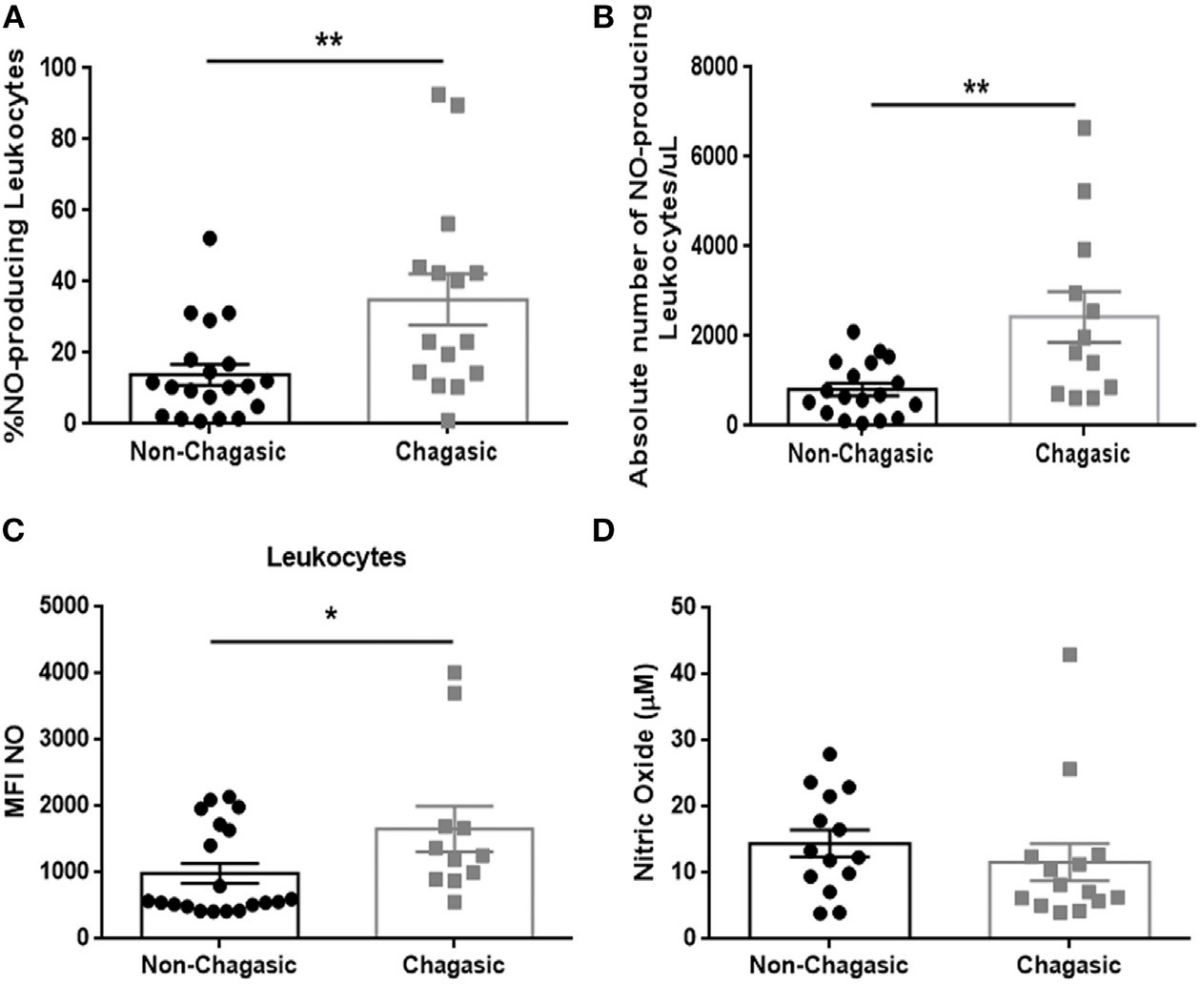
**Chagasic patients exhibit a higher number and frequency of nitric oxide-producing leukocytes**. **(A)** Percentage and **(B)** absolute number of nitric oxide (NO)-producing leukocytes from chagasic patients (*n* = 15) and non-chagasic donors (*n* = 19). **(C)** Mean fluorescence intensity of NO in leukocytes from chagasic patients (*n* = 11) and non-chagasic donors (*n* = 20). **(D)** Serum nitric oxide levels in seropositive patients (*n* = 14) and seronegative donors (*n* = 14) (*p < 0.05 and **p < 0.01).

Seropositive patients showed an increased percentage and absolute number of NO-producing leukocytes (Figures 2A,B), with higher production of NO per leukocyte (Figure 2C), but no significant differences were detected in levels of plasma NO in comparison with seronegative subjects (Figure 2D). The infection of peripheral blood from seronegative and seropositive patients with trypomastigotes (Tulahuen strain) significantly increased the percentage of NO- and ROS-producing leukocytes (Figure S2 in Supplementary Material).

### CD8+ T Lymphocytes from Chagasic Patients Exhibited High Levels of Nitrated Tyrosine Residues

**Figure 3:**
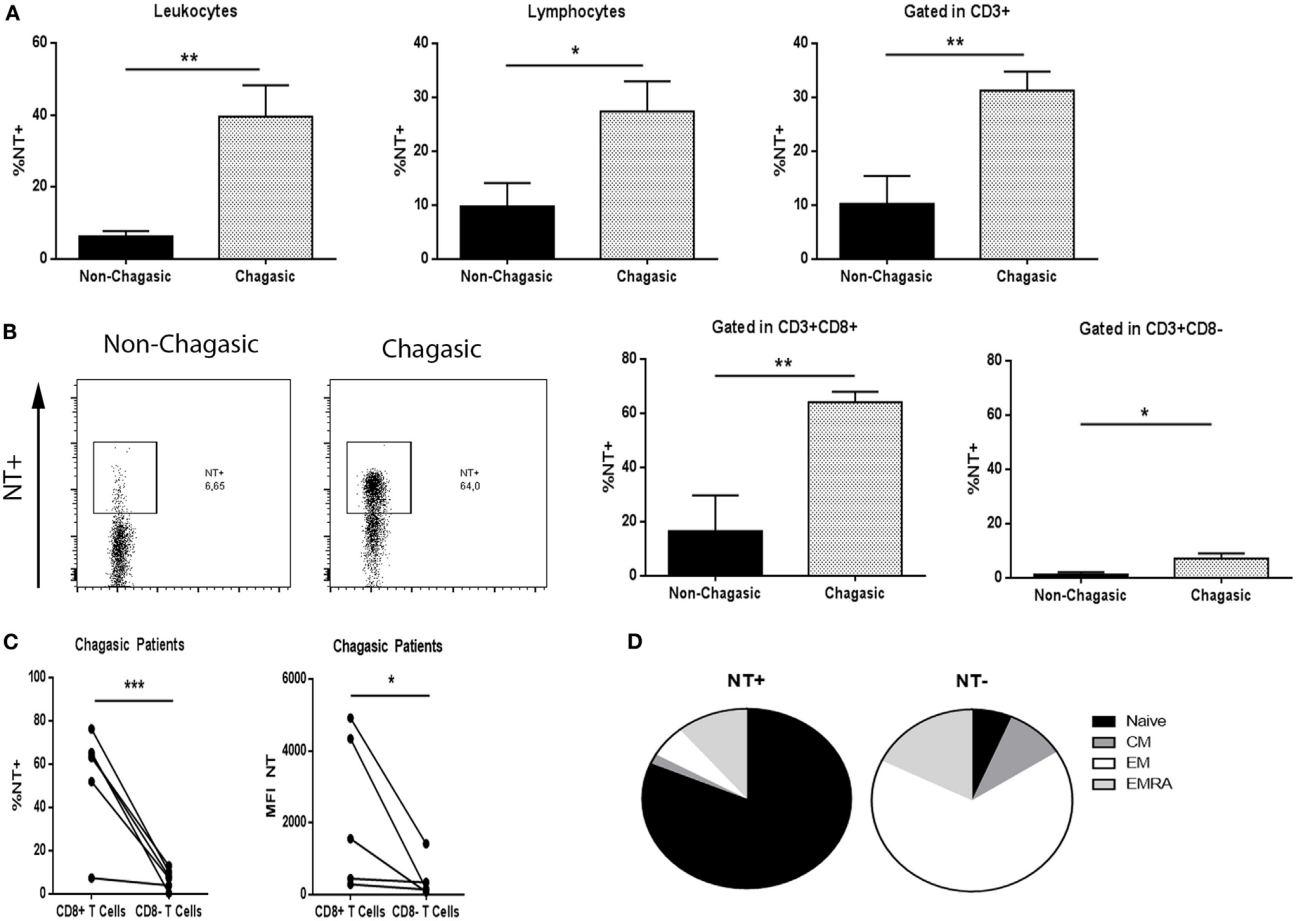
**CD8+ T lymphocytes from chagasic patients present high tyrosine nitration**. **(A)** Percentage of nitrated (NT) leukocytes, lymphocytes, and T cells from seropositive patients (*n* = 6) and seronegative donors (*n* = 6). **(B)** Representative dot plot of tyrosine nitration on cytotoxic T-cell surface and percentage of NT+CD3+CD8+ and NT+CD3+CD8− lymphocytes from chagasic patients (*n* = 6) and non-chagasic donors (*n* = 6). **(C)** Percentage of NT+CD3+CD8+ and NT+CD3+CD8− and mean fluorescence intensity of NT in CD3+CD8+ and CD3+CD8− cells from chagasic patients (*n* = 5) (*p < 0.05, **p < 0.01, and ***p < 0.001). **(D)** Frequency of naïve, central memory, effector memory (EM), and effector memory RA gated in NT+ or NT− in CD8+ cells from chagasic patients (*n* = 4) (naïve NT+ vs. naïve NT−, *p* < 0.0001; EM NT+ vs. EM NT−, *p* < 0.0001).

Non-chagasic individuals exhibited significantly lower tyrosine nitration (NT) of leukocytes, lymphocytes, and T cells than chagasic patients (Figure 3A). In particular, chagasic patients showed more NT+CD8+ T lymphocytes compared to non-chagasic donors (Figure 3B). Although nitration also increased in CD8− T cell population from seropositive patients, a main effect was observed in CD8+ T lymphocytes compared to CD8− T cells in this group of patients (Figure 3C). Among NT+ cytotoxic cells from Chagas patients, we have identified naïve cells as the main subpopulation that undergoes tyrosine nitration (NT+ vs. NT− 81.45 ± 3.00 vs. 6.50 ± 1.61%). On the contrary, the majority of NT− cells were EM CD8+ T cells (NT+ vs. NT− 5.73 ± 1.22 vs. 66.33 ± 4.46%) (Figure 3D).

### Cytotoxic T Cells from Chagas Patients Were Less Functional

**Figure 4:**
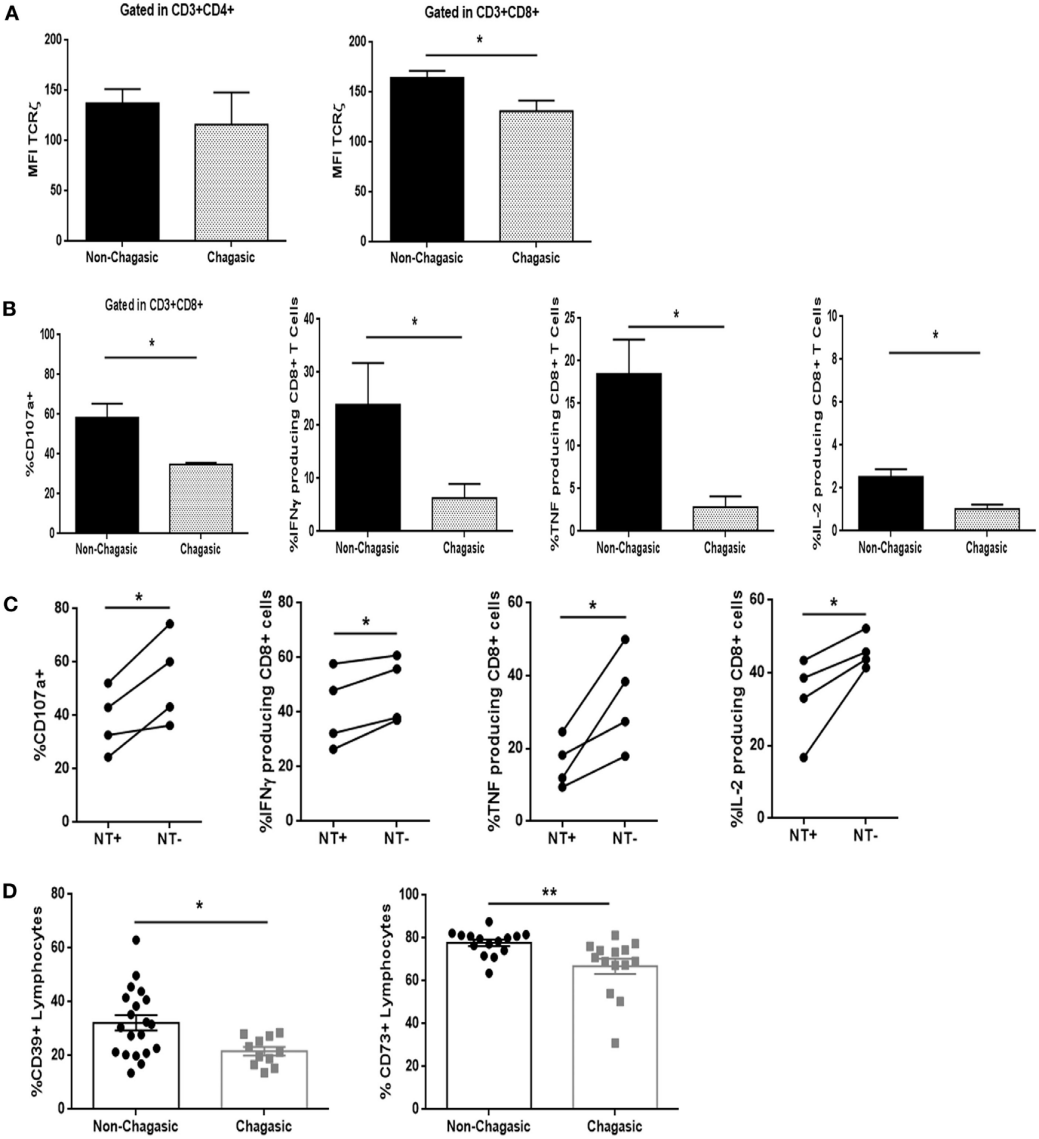
**Cytotoxic T cells from chagasic patients are less functional than those from healthy individuals**. **(A)** Mean fluorescence intensity of TCRζ-chain in CD3+CD4+ and CD3+CD8+ cells; **(B)** frequency of CD107a+ cells, IFNγ, TNF, and IL-2-producing CD8+ T lymphocytes from chagasic patients (*n* = 5) and seronegative donors (*n* = 5) after PMA/ionomycin stimulation. **(C)** Frequency of CD107a+ cells, IFNγ, TNF, and IL-2-producing NT+ and NT− gated in CD8+ T lymphocytes from chagasic patients (*n* = 4) after anti-CD3+ anti-CD28 stimulation. **(D)** Percentage of CD39+ and CD73+ leukocytes from seropositive (*n* = 20) and seronegative (*n* = 14) individuals (*p < 0.05 and **p < 0.01).

Even though CD4+ T lymphocytes from seropositive and seronegative individuals showed similar levels of expression of the TCRζ chain, the cytotoxic T cells exhibited a diminished amount of TCRζ in chagasic patients, as compared to non-chagasic donors (Figure 4A). In line with these observations, CD8 T cell population from seropositive patients showed lower frequency of CD107a+ cells, as well as IFNγ, TNF, and IL-2-producing cells after stimulation with PMA/Ionomycin (unspecific stimuli) in comparison with the same population from seronegative donors (Figure 4B). Furthermore, after anti-CD3/anti-CD28 stimulation (*via* TCR), the percentage of CD8+ T cells from Chagas patients positive for CD107a, IFNγ, TNF, and IL-2 was significantly diminished in NT+ cells compared to NT− cells (Figure 4C). The same behavior was observed when we compared the functionality of nitrated vs. no nitrated CD8+ T cells within the EM–EMRA populations (Figure S3 in Supplementary Material). These results indicated that nitrated cytotoxic T cells from chagasic patients were less functional than non-nitrated population. Moreover, chagasic patients showed a decreased percentage of CD39+ and CD73+ lymphocytes compared to control donors (Figure 4D). However, frequency of CD8+ T cells expressing Tim-3, PD-1, or CTLA-4 was no different between chagasic patients and seronegative donors (Figure S4 in Supplementary Material). The results suggest that although cytotoxic T cells are less functional in seropositive subjects, they appear not to be a classically exhausted population since the expression of inhibitory receptors was not upregulated compared to CD8+ T cells from seronegative subjects.

### IL-1β Plasma Levels Were Increased in Seropositive Patients

**Figure 5:**
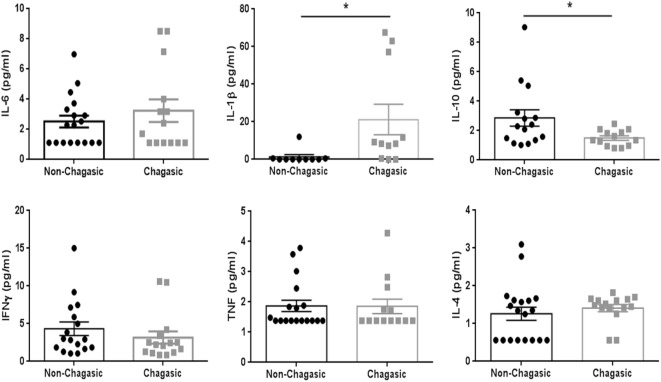
**IL-1β plasma levels are higher in seropositive patients than in seronegative donors**. Plasma levels of IL-6, IL-10, IFNγ, TNF, and IL-4 (assayed by a panel of capture beads) and IL-1β (evaluated by enzyme-linked immunosorbent assay) in chagasic patients (*n* = 18) and non-chagasic donors (*n* = 15) (*p < 0.05).

The cytokine plasma levels were assayed using a panel of capture beads. In seronegative and seropositive individuals, the levels of IL-6, IFNγ, TNF, and IL-4 were similar. However, IL-1β levels were higher, and the amount of IL-10 was lower in plasma from chagasic people compared with control donors (Figure 5).

### IL-6 Prevented CD8+ T Cell Nitration and Apoptosis Susceptibility and Decreased IL-1β Production by Infected PBMCs

**Figure 6:**
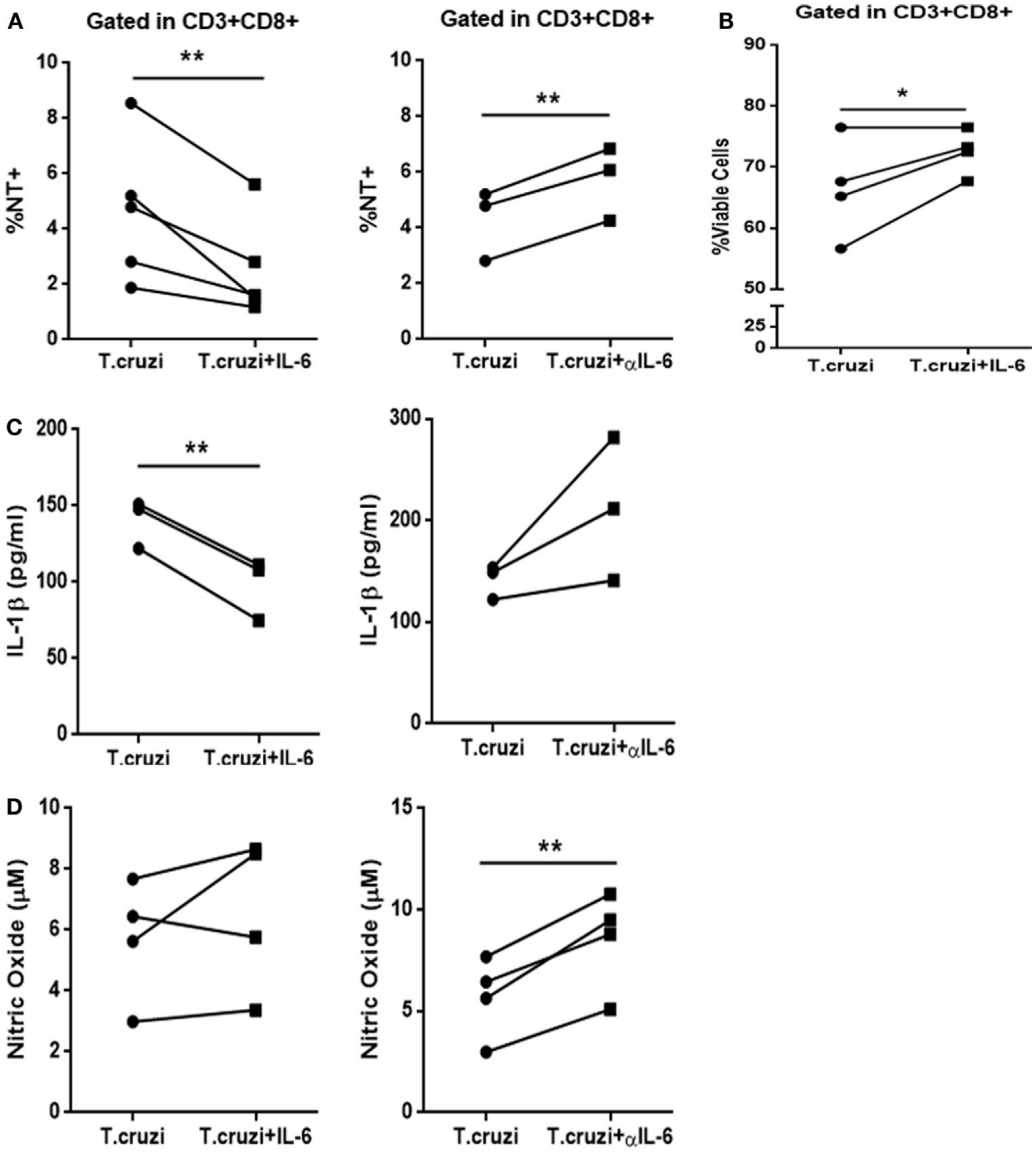
**IL-6 prevents nitration, increases survival of CD3+CD8+ cells, and decreases IL-1β levels after infection**. **(A)** Percentage of NT+CD3+CD8+ cells in infected peripheral blood mononuclear cells (PBMCs) from non-chagasic (*n* = 5) donors after 48 h of stimulation with IL-6 (*Trypanosoma cruzi* + IL-6) or αIL-6 (*T. cruzi* + αIL-6) or medium alone (*T. cruzi*). **(B)** Percentage of viable CD3+CD8+ cells infected with *T. cruzi* (*T. cruzi*) or infected and stimulated with IL-6 (*T. cruzi* + IL-6). **(C)** IL-1β levels and **(D)** nitric oxide levels in culture supernatants of infected PBMCs after 48 h of stimulation with IL-6 (*T. cruzi* + IL-6) or αIL-6 (*T. cruzi* + αIL-6) or medium alone (*T. cruzi*) (*p < 0.05 and **p < 0.01).

**Figure 7:**
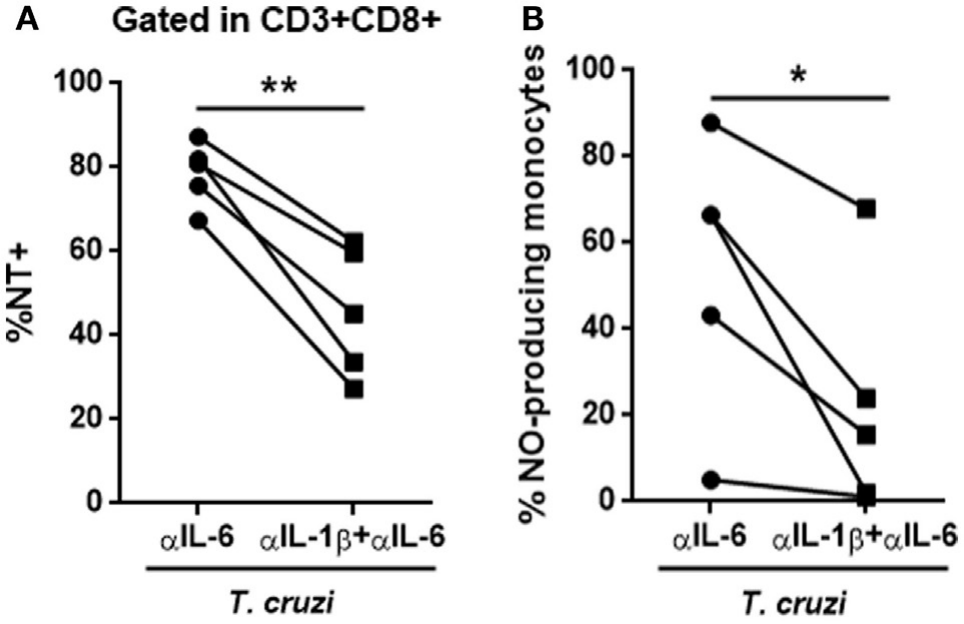
**The blockage of IL-6 and IL-1β reverts the phenotype induced by anti-IL-6**. **(A)** Percentage of NT+CD8+ T cells and **(B)** NO-producing monocytes (CD14+) in *in vitro*-infected peripheral blood mononuclear cells from seronegative (*n* = 5) subjects after 48 h of stimulation with anti-IL-6 or anti-IL-6 + anti-IL-1β (*p < 0.05 and **p < 0.01).

The IL-6 stimulation of *in vitro-*infected PBMCs obtained from healthy donors blunted *T. cruzi*-induced NT in CD3+CD8+ cells. Conversely, the treatment of these cultures with a neutralizing antibody specific for IL-6 (αIL-6) increased the percentage of *T. cruzi*-induced NT in this subpopulation (Figure 6A). Moreover, IL-6 treatment rescued infected CD8+ T cells from apoptosis (Figure 6B). Concomitantly, while IL-6 stimulation diminished the levels of IL-1β in culture supernatants from *T-cruzi*-infected PBMCs, αIL-6 treatment increased the release of IL-1β (Figure 6C). Furthermore, although IL-6 stimulation did not affect NO production by *T. cruzi*-infected PBMCs, the blockage of IL-6 significantly increased *T. cruzi*-induced NO production (Figure 6D). Additionally, IL-6 stimulation diminished NT in infected CD3+CD8+ cells from peripheral blood of chagasic patients (Figure S5 in Supplementary Material). In accordance, we found that blocking IL-6 and IL-1β significantly diminished the percentage of NT+CD8+ T cells from *in vitro-*infected PBMCs in comparison with cultures incubated with anti-IL-6 alone (Figure 7A). Moreover, the percentage of NO-producing monocytes significantly diminished when both cytokines were blocked compared with the inhibition of IL-6 alone (Figure 7B).

## Discussion

Multiple immunological effector mechanisms are critical for resolving *T. cruzi* infection, but considering that this parasite invades and replicates in essentially all types of mammalian cells, T cells and monocytes/macrophages are particularly important for controlling the infection. As with other intracellular parasites, *T. cruzi* antigens are processed and presented on MHC-class I molecules, leading to the recognition of parasite components by CD8+ T cells. In this regard, the study of the induction of CD8+ T cell-mediated protective immunity has become a center of intense research efforts to find control measures and prophylactic tools that could be used to produce effective therapeutic vaccines. This study shows that chronic *T. cruzi* infection leads to a significant nitration of T lymphocytes, mainly of the CD8+ T cell subset. The increased tyrosine nitration was associated with impaired effector functions and a significant fall in the number and percentage of circulating CD8+ T cells in chronic Chagas patients.

Among the reactive oxygen and nitrogen species mediating T-cell suppression is peroxynitrite, one of the most potent oxidants in the body. The hyperproduction of peroxynitrite is associated with nitration of the surface proteins in T cells (11, 12). Regarding CD8+ T cells, nitration of tyrosines within the TCR/CD8 complex disrupts the binding of the specific peptide-MHC dimers to CD8 molecules, which results in the inability of this T cell subset to bind MHC (18, 19). Furthermore, peroxynitrites also inhibit TCR signaling by preventing the association of CD3-ζ with the TCR. Our group has reported that *T. cruzi* induces an increase in splenic NT+CD8+ and NT+CD4+ T cells from infected mice by performing confocal and flow cytometric analysis of immunofluorescence staining (13). In this work, we found that the increased nitration of CD8+ T cells was associated with a lower capacity for activation (TCRζ expression), a diminished production of cytokines (IFNγ, TNF, and IL-2) and deactivation of cytotoxic functions (CD107a expression) in this cell population. Strikingly, cellular nitration seems to be reversible, since the incubation of infected leukocytes from seropositive patients with IL-6 significantly diminished the percentage of NT+ lymphocytes.

Leukocytes from chagasic patients significantly increased the production of NO, which correlated with increased susceptibility of CD8+ T cells to undergo spontaneous apoptosis. NO-mediated suppression of T-cell activation does not seem to be mediated by events triggered by TCR recognition but, instead, with the signaling cascade that is downstream of IL-2 (20). In human T cells, NO affects the stability of IL-2 mRNA and the release of IL-2. In this sense, NO negatively regulates intracellular signaling proteins either directly, by S-nitrosylation of crucial cysteine residues, or indirectly, by activation of cyclic-GMP-dependent protein kinase (21). Furthermore, while NO also sensitizes cells to Fas-L-mediated apoptosis (22), the anti-apoptotic molecule Bcl-2 diminished the susceptibility to NO-induced apoptosis (23). In line with these reports, we found that CD8+ T cells from seropositive individuals showed a significantly decreased expression of Bcl-2 concomitant with the significant diminution in their viability. The increased susceptibility to apoptosis was in accordance with the diminution in the percentage as well as absolute number of total T cells at the expense of diminution of CD8+ T cells but not of T helper cells or B cells.

It was reported that NO levels, as measured by Griess reagent assay, were not significantly increased in the plasma of seropositive subjects compared with those in seronegative samples. However, the plasma level of 3-nitrotyrosine (NT), was increased in seropositive subjects (24). The results indicated that seropositive subjects with Chagas disease are exposed to increased nitrosative stress. In total agreement with this report, we observed no significant increase in plasma levels of nitrate/nitrite of seropositive subjects compared with seronegative donors. Nevertheless, the leukocytes from chagasic patients significantly increased the production of NO, suggesting that NO produced by leukocytes, rather than soluble NO, may contribute to increased nitrosative stress in patients with Chagas disease, evidencing the importance of cell-to-cell contact for NO inhibitory effector functions (25, 26). Although tyrosine nitration constitutes one of the mechanisms employed by MDSC to suppress T cell response through cell-to-cell contact, it may also be dependent on the activity of myeloperoxidase, secreted by monocytes and polymorphonuclear neutrophils (27).

In accordance with our recent observations, while IL-1β plasma levels were significantly increased in chagasic patients, the stimulation of *in vitro*-infected PBMCs with IL-6 significantly diminished the production of IL-1β. Moreover, IL-6 diminished the nitration rate and improved the survival of CD8+ T cells. In agreement with this result, we have previously shown that IL-6 acts as a survival factor for infected target cells (28, 29). We have observed that IL-6 regulates IL-1β-induced NO production, and that excessive oxidative stress accounts for the increased mortality of *T. cruzi*-infected IL6KO mice (unpublished results). The anti-inflammatory action of IL-6 appears to be central to controlling cardiac and systemic oxidative stress, promoting cellular rescue against apoptosis, and protecting infected IL-6-deficient mice against death. In this model, this was clearly illustrated by the fact that the percentage of nitrated CD8+ T cells observed after blocking IL-6 was significantly diminished when anti-IL-6 was combined with anti-IL-1β. In accordance, IL-6 stimulation of peripheral blood cells from chagasic patients induced a significant diminution of nitration of CD8+ T cells. Our results strongly suggest that IL-6 could represent a key factor for regulating the nitration of proteins on human cytotoxic T cells.

One potential regulatory system that could have a role in the IL-6-induced anti-inflammatory effects is the extracellular levels of adenosine. *T. cruzi* infection of cardiac tissue induces an influx of immune cells that consume large quantities of oxygen. Ischemic cells rapidly respond to the hypoxic and inflammatory environment by releasing ATP (normally present within cardiomyocytes in millimolar concentrations). Once in the extracellular environment, ATP is converted to AMP and then to adenosine by the CD39 and CD73 ectoenzymes, respectively (30). In pathological conditions, high levels of ATP act as a pro-inflammatory danger signal, activating the inflammasome that processes pro-IL-1β into mature IL-1β (31, 32). In this sense, it has been suggested that CD39 expression may contribute to dampening the ongoing inflammatory processes and/or rescue the cells from ATP-induced apoptosis/necrosis (33). Ultimately, adenosine exerts potent anti-inflammatory effects on different immune cell types. Recently, we reported that purinergic signaling is clue in regulating the immune response to experimental *T. cruzi* infection. The temporal pharmacological inhibition of CD73 during the early acute phase of the infection induces microbicidal mechanisms, with the concomitant reduction in cardiac parasite load, improving the outcome of chronic cardiomyopathy (34). In agreement with a previous report that indicated CD73 expression on T cells is downregulated in chronic HIV infection (35), in this work, we observed lower expression of CD39 and CD73 on fresh explanted lymphocytes from chagasic patients compared to seronegative donor. Thus, it is plausible that the downregulation of ATP catabolic enzymes could be involved in the increased plasma IL-1β levels in chagasic patients.

In summary, the results of this study show that chronic *T. cruzi* infection leads to a decrease in the number and percentage of total circulating CD8+ T cells and that NO produced by leukocytes may contribute to lowering the effector function of these cells, which ultimately may results in an inefficient control of parasite replication. The findings also suggest that IL-6 could be a key factor to improve CD8+ T cell activation and survival.

## Author Contributions

Conceived and designed the experiments: LS, MR, and MA. Performed the experiments: LS, LV, NE, MR, and NP. Analyzed the data: LS, LV, NE, MR, NP, NS, MV, GB, SG, AM, and MA. Patients handling and human samples: LV, NS, MV, GB, and AM. Wrote the paper: LS and MA.

## Conflict of Interest Statement

The authors declare no competing financial interests. They did not receive payment or services from a third part for any aspect of the submitted work. The reviewer CL and handling Editor declared their shared affiliation, and the handling Editor states that the process nevertheless met the standards of a fair and objective review.
