# Supplementary material for: IL-6 Improves the Nitric Oxide-Induced Cytotoxic CD8+ T Cell Dysfunction in Human Chagas Disease
Source: Front Immunol. 2016 Dec 23;7:626. doi: 10.3389/fimmu.2016.00626 (PMC5179535; doi:10.3389/fimmu.2016.00626)
Supplement: Supplementary file 1 [file Image_1.PDF]

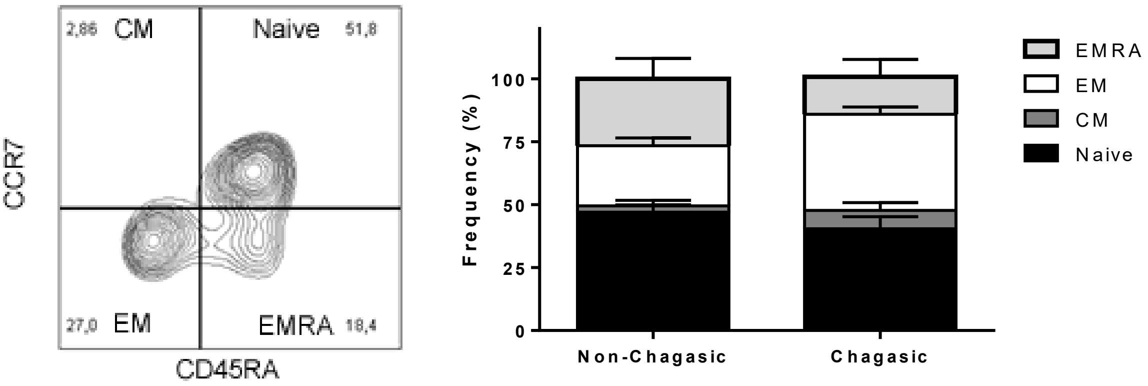

Supplementary Figure 1: Chagasic patients showed increased percentage of effector memory CD8+ T cells. (A) Representative dot plots showing CD45RA and CCR7 expression in CD8 T-cell subsets (naïve, central memory [CM], effector memory [EM] and EMRA). (B) Frequency of CD8+ T cell subsets in chagasic patient and non-chagasic donors. (% EM in non-chagasic donors vs %EM in chagasic subjects p = 0.047).
