# Supplementary material for: IL-6 Improves the Nitric Oxide-Induced Cytotoxic CD8+ T Cell Dysfunction in Human Chagas Disease
Source: Front Immunol. 2016 Dec 23;7:626. doi: 10.3389/fimmu.2016.00626 (PMC5179535; doi:10.3389/fimmu.2016.00626)
Supplement: Supplementary file 2 [file Image_2.PDF]

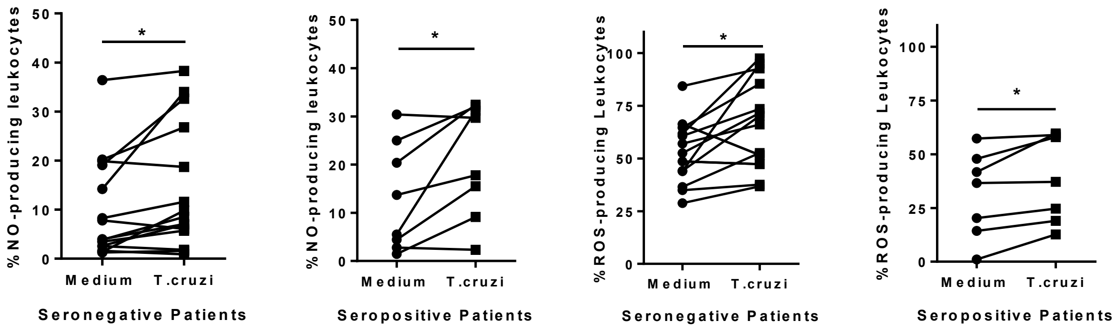

Supplementary Figure 2: *T.cruzi* infection induces reactive oxygen species and nitric oxide production

Percentage of nitric oxide and ROS-producing leukocytes post-*T.cruzi* cultured peripheral blood from chagasic patients (n = 8) and non-chagasic donors (n = 15) after 24 h. * $p < 0.05$.
