# Supplementary material for: IL-6 Improves the Nitric Oxide-Induced Cytotoxic CD8+ T Cell Dysfunction in Human Chagas Disease
Source: Front Immunol. 2016 Dec 23;7:626. doi: 10.3389/fimmu.2016.00626 (PMC5179535; doi:10.3389/fimmu.2016.00626)
Supplement: Supplementary file 3 [file Image_3.PDF]

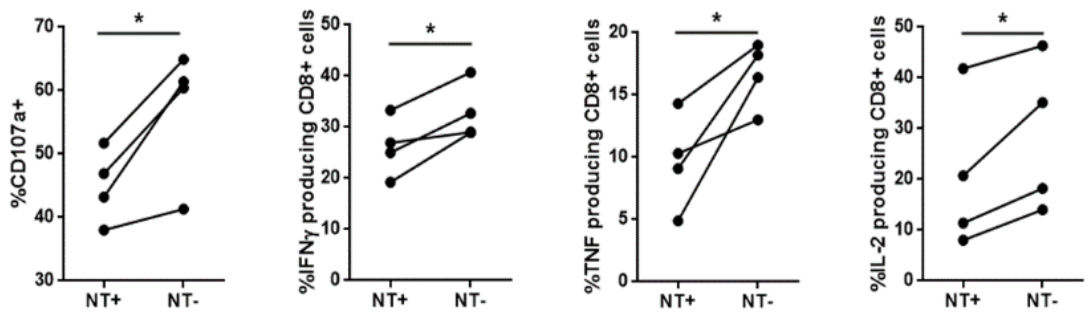

Supplementary Figure 3: EM-EMRA NT+ cytotoxic T cells from chagasic patients are less functional than EM-EMRA NT-cells

Frequency of CD107a+ cells, IFNγ, TNF and IL-2–producing NT+ and NT- CD8+ T lymphocytes from chagasic patients (n = 4) gated in CCR7- (EM-EMRA subpopulation) after anti-CD3+anti-CD28 stimulation. * $p < 0.05$.
