# Supplementary material for: IL-6 Improves the Nitric Oxide-Induced Cytotoxic CD8+ T Cell Dysfunction in Human Chagas Disease
Source: Front Immunol. 2016 Dec 23;7:626. doi: 10.3389/fimmu.2016.00626 (PMC5179535; doi:10.3389/fimmu.2016.00626)
Supplement: Supplementary file 4 [file Image_4.PDF]

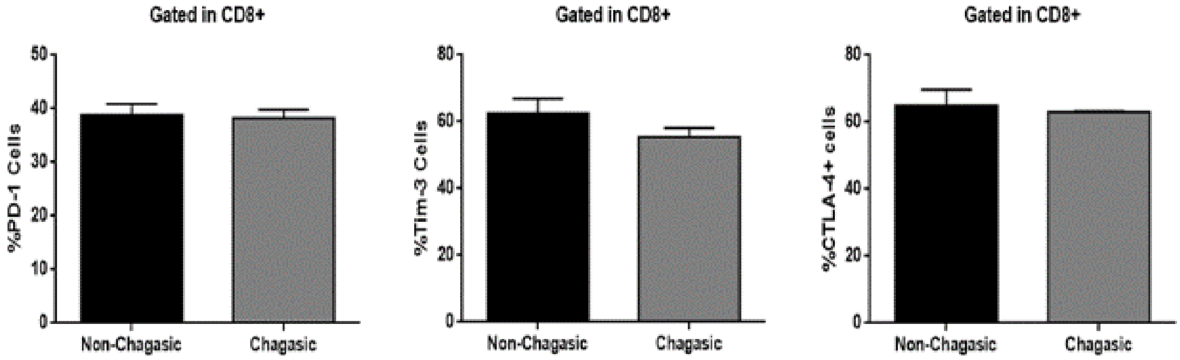

Supplementary Figure 4: The percentage of exhaustion markers in CD8+ cells are not different between chagasic and non-chagasic patients. Frequency of PD-1, Tim-3 and CTLA-4 in CD8+ cells from chagasic patients (n = 4) and non-chagasic donors (n = 4) after anti-CD3 and anti-CD28 stimulation.
