# Supplementary material for: IL-6 Improves the Nitric Oxide-Induced Cytotoxic CD8+ T Cell Dysfunction in Human Chagas Disease
Source: Front Immunol. 2016 Dec 23;7:626. doi: 10.3389/fimmu.2016.00626 (PMC5179535; doi:10.3389/fimmu.2016.00626)
Supplement: Supplementary file 5 [file Image_5.PDF]

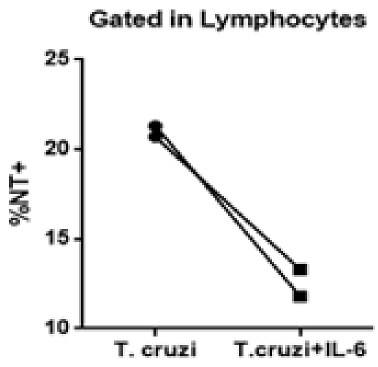

Supplementary Figure 5: IL-6 diminishes nitration in CD3+CD8+ from chagasic patients

Percentage of NT+CD3+CD8+ cells in *T.cruzi* and *T. cruzi*+IL-6-cultured peripheral blood from chagasic patients (n = 2) after 24 h
